# Imaging elliptically polarized infrared near-fields on nanoparticles by strong-field dissociation of functional surface groups

**DOI:** 10.1140/epjd/s10053-022-00430-6

**Published:** 2022-06-27

**Authors:** Philipp Rosenberger, Ritika Dagar, Wenbin Zhang, Ana Sousa-Castillo, Marcel Neuhaus, Emiliano Cortes, Stefan A. Maier, Cesar Costa-Vera, Matthias F. Kling, Boris Bergues

**Affiliations:** 1 Department of Physics, Ludwig-Maximilians-Universität Munich, D-85748 Garching, Germany; 2 Max Planck Institute of Quantum Optics, D-85748 Garching, Germany; 3 State Key Laboratory of Precision Spectroscopy, East China Normal University, Shanghai, 200241 China; 4 Chair in Hybrid Nanosystems, Nanoinstitute Munich, Königinstrasse 10, Faculty of Physics LMU Munich, 80539 Munich, Germany; 5 Department of Physics, Imperial College London, London, SW7 2AZ UK; 6 School of Physics and Astronomy, Monash University, Clayton Victoria, 3800 Australia; 7 Departamento Fisica, Escuela Politecnica Nacional, 170109 Quito, Ecuador; 8 SLAC National Accelerator Laboratory, Menlo Park, CA 94025 USA; 9 Applied Physics Department, Stanford University, Stanford, CA 94305 USA

## Abstract

**Abstract:**

We investigate the strong-field ion emission from the surface of isolated silica nanoparticles aerosolized from an alcoholic solution, and demonstrate the applicability of the recently reported near-field imaging at 720 nm [Rupp et al., Nat. Comm., 10(1):4655, 2019] to longer wavelength (2 $\mu$m) and polarizations with arbitrary ellipticity. Based on the experimental observations, we discuss the validity of a previously introduced semi-classical model, which is based on near-field driven charge generation by a Monte-Carlo approach and classical propagation. We furthermore clarify the role of the solvent in the surface composition of the nanoparticles in the interaction region. We find that upon injection of the nanoparticles into the vacuum, the alcoholic solvent evaporates on millisecond time scales, and that the generated ions originate predominantly from covalent bonds with the silica surface rather than from physisorbed solvent molecules. These findings have important implications for the development of future theoretical models of the strong-field ion emission from silica nanoparticles, and the application of near-field imaging and reaction dynamics of functional groups on isolated nanoparticles.

**Graphical abstract:**

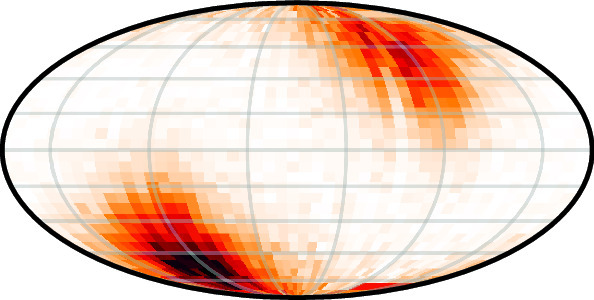

**Supplementary Information:**

The online version contains supplementary material available at 10.1140/epjd/s10053-022-00430-6.

## Introduction

Chemical processes unfolding on the surface of nanoparticles are at the heart of numerous applications in biology  [1], catalysis  [2], atmospheric science  [3], and electrochemistry  [4]. As the chemical composition of the nanoparticle surface is a determining factor for these processes, the ability to image the surface composition and the chemical reaction landscapes with nanoscopic spatial resolution, and potentially femtosecond temporal resolution would be highly interesting. Strong-field ionization of the surface combined with charged particle imaging techniques has the potential to facilitate both femtosecond temporal and nanoscopic spatial resolution. Imaging electron emission from nanoparticles in strong laser fields has been instrumental in demonstrating the spatiotemporal control of surface fields  [5, 6], the measurement of attosecond photoemission delays  [7] as well as laser-induced metallization  [8]. However, the spatial resolution of the method, which relied on field-driven backscattered electrons, was limited by charge-interactions and scattering dynamics. Furthermore, only a small fraction of all emitted electrons, namely the ones close to their energetic cutoff, were suitable for imaging the near-field distributions  [9–11]. Moreover, the comparatively low ionization potential of the nanoparticles’ bulk as compared to that of most molecules makes this method insensitive to the presence of molecules adsorbed on the surface. Strong-field emission of ions, in contrast, provides a sensitive probe for photo-induced reactions of surface molecules, as demonstrated with the recently introduced reaction nanoscopy technique  [12]. In particular, the method facilitates the imaging of the enhanced near-fields around dielectric nanoparticles  [12], while also providing information about their morphology  [13]. Recently, reaction nanoscopy unveiled the laser-induced formation of trihydrogen cations on water-covered silica nanoparticles, thereby extending its application to surface photochemistry  [14].

Reaction nanoscopy enables the study of laser-generated ions emitted from a variety of nanoparticle materials aerosolized from a solution. Three-dimensional ion momenta are reconstructed from a time-of-flight and position measurement. Previous studies have revealed that the measured ion momentum distribution closely follows the distribution of the optical near-field on the nanoparticle surface  [12, 13]. So far, reaction nanoscopy has been demonstrated for linearly polarized fields at wavelengths of 720 nm  [12] and 1030 nm  [13, 14]. In addition to the near-field, reaction nanoscopy also allows a mapping of chemical reaction yields on a nanoparticle surface with nanometer resolution. While reaction nanoscopy has proven to be a powerful imaging technique in the short wavelength range of the near infrared region and for linear laser polarization, its applicability to a wider range of laser parameters has remained an open question. It is not clear, for instance, whether the use of a different polarization or wavelength has a detrimental effect on the imaging capability. Since strong-field phenomena are known to be strongly dependent on the wavelength and the polarization of the driving field  [15–18], this would not be unexpected.

In addition, the exact role of the solvent and the nature of the solvent–silica interface has remained unclear up until now. While fragments of solvent molecules were proposed to yield the main contribution to the measured ion signal, detailed experimental investigations have been lacking so far.

In the present study, we investigate the applicability of reaction nanoscopy to different polarizations and its extension toward the mid-infrared regime, at a wavelength of 2 $\mu$m. While most previous reaction nanoscopy studies have been focusing on proton emission, we discuss contributions of heavier fragments from induced molecular processes on the surface of the nanoparticles. Our findings shed light on the surface composition of the nanoparticles and clarify the role of alcoholic solvents in the observed ion spectra.

**Fig. 1:**
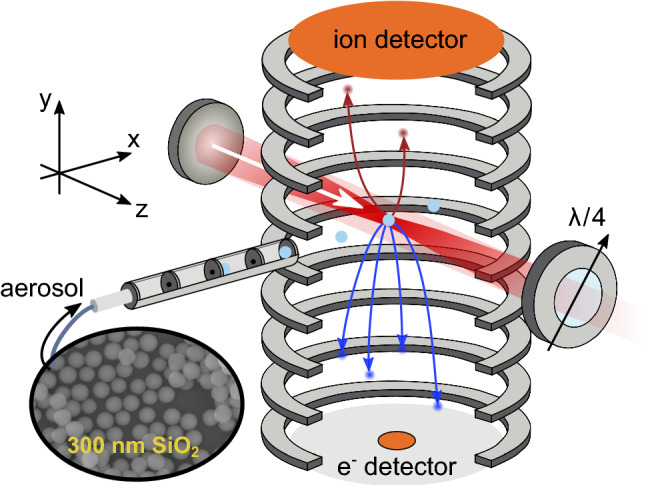
Schematic depiction of the setup. A dried aerosol of silica nanoparticles (cyan) with a diameter of 300 nm is collimated by an aerodynamic lens and introduced into the reaction nanoscope. Laser pulses are focused into the stream of nanoparticles. Laser-generated electrons (blue) and ions (red) are detected by a channeltron and a delay-line detector, respectively. The polarization state of the laser pulses is controlled by a quarter wave plate. The coordinate system indicated here is used throughout the work

**Fig. 2:**
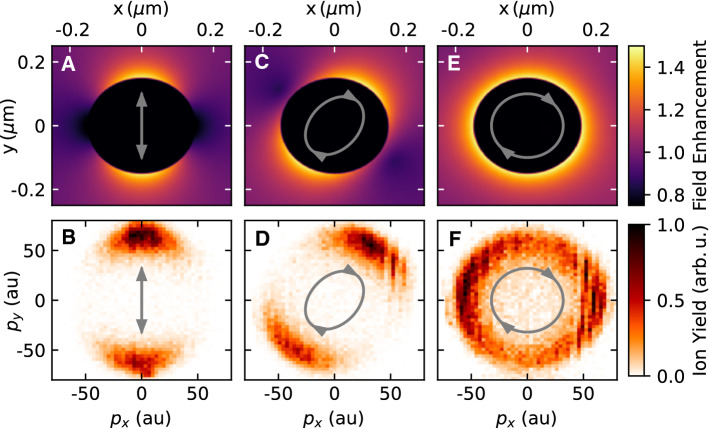
Comparison between the field enhancement in the plane $z=0$ (panels A, C, E) and the projection of the proton momenta onto the *xy*-plane (panels B, D, F) for 300 nm silica nanoparticles. The gray symbols indicate the polarization state for each panel. First column (panels A,B): results for linear polarization (waveplate angle: 0$^\circ$). Second column (panels C, D): results for an elliptic polarization (waveplate angle: 35$^\circ$). Third column (panels E,F): results for circular polarization (waveplate angle: 45$^\circ$). The vertical streaks in panels D and F are caused by artifacts of the delay line detection. The pulse energy is 18 $\mu$J in all cases, corresponding to a peak intensity of about $2\times 10^{13}$ W/cm$^2$ in the linearly polarized case

## Experimental methods

### Laser system

Laser pulses with a central wavelength of 2 $\mu$m, a pulse duration of 25 fs and a maximum pulse energy of 100 $\mu$J were generated via a custom optical parametric chirped-pulse amplifier system with a repetition rate of 100 kHz  [19]. The pulses were attenuated by a combination of a broadband half wave plate and wire grid polarizer, and their ellipticity was adjusted with a broadband quarter wave plate (waveplates: B. Halle Nachfl. GmbH, RAC 6L series).

### Reaction nanoscope

The laser pulses were directed into the ultra-high vacuum chamber of the reaction nanoscope (Fig. 1) where they were back-focused with a spherical silver mirror ($f=$75 mm) onto a stream of aerosolized silica nanoparticles, reaching intensities on the order of 10$^{13}$ W/cm$^2$. The intensity was estimated from the pulse duration and focal profile. Upon interaction with the laser pulses, the nanoparticle surface is ionized, leading to a large number of free electrons and a typically small number of ions originating from the dissociation of surface adsorbents. A homogeneous electric field applied across the spectrometer accelerates the electrons and ions toward their respective detectors located at opposing sides of the spectrometer. Ions are detected with a time- and position-sensitive detector consisting of an 80 mm multi-channel plate stack in combination with a delay-line anode (DLD80, RoentDek Handels GmbH), while the coincident detection of the electrons is facilitated by a channeltron electron multiplier. The three-dimensional ion momenta are calculated from the measured time-of-flight (TOF) information and impact position on the position-sensitive detector. The electrons signal is read out by a fast analog-to-digital converter (fADC4, RoentDek Handels GmbH) and digitally time-integrated. Filtering on large integrated electron signals allows us to single out nanoparticle ionization events and efficiently reduce the contribution of background-gas ionization. For more details on the reaction nanoscopy technique, see Ref. [12].

### Nanoparticle delivery

In the present study, we used 300 nm spherical SiO$_2$ nanoparticles dispersed in ethanol (Fisher Chemical, $\ge$99%), methanol (Thermo Scientific Alfa Aesar, 99%) or methanol-d4 (ACROS Organics, 99.6 atom-% D), respectively. The solutions were aerosolized employing an aerosol generator (TSI inc., model 3076). Argon was used as an aerosolization and carrier gas for the transmission of the silica nanoparticles to the reaction nanoscope. Excess solvent was removed from the aerosol by a counter-flow membrane dryer (PermaPure MD-700-48S). A small-aperture impactor (TSI inc., part no. 1035900) was used to reduce the number of clusters in the stream. The nanoparticle beam was collimated by an aerodynamic lens  [20] and sent into the reaction nanoscopy after passing three differential pumping stages.

### Nanoparticle synthesis

The silica nanoparticles with diameter of 300 nm were synthesized by a modified Stöber method  [21]. The reagents and materials of tetraethoxysilane (TEOS, $\ge$98%), ethanol (EtOH, absolute), and ammonia solution (NH$_4$OH, 27 wt % in water) were purchased from Sigma-Aldrich. Ultrapure water with a resistivity of 18.2 M$\Omega$cm and a total organic content of $\le$3 ppb was obtained from a Millipore Milli-Q system. For the particle synthesis, 1.7 ml of TEOS solution was added to a solution containing ethanol (18.12 ml), ammonium hydroxide (1.96 ml), and water (3.21 ml). This mixture was stirred at room temperature for 2 h and then purified by three centrifugation–redispersion cycles with ethanol (6000 rpm, 10 min). The size and morphology of the silica nanoparticles were characterized using a transmission electron microscope (TEM, JEOL1020) operating at 80 kV. A standard deviation of $6\%$ for the size distribution of the nanoparticles was obtained from the electron microscope image. The procedure is described in the supplementary material. All samples were stored in ethanol after cleaning. In order to replace the ethanol by the other solvents, we carried out the same centrifugation–redispersion cycles as in the purification step after the synthesis. Before using the samples in the experiment, they were further diluted to a concentration of 1 g/l in order to minimize the amount of clusters in the nanoparticle stream, as verified by morphology analysis with the reaction nanoscope  [13].

## Theoretical model

The model used here is very similar to previously introduced theoretical descriptions of the interaction between strong optical fields and dielectric nanoparticles  [12, 13]. When light scatters from a spherical dielectric nanoparticle, the electric field in its vicinity is enhanced in certain regions. The exact location and shape of those regions is given by the Mie solution of Maxwell’s equations  [22]. If a strong laser is used as the light source, it can drive nonlinear processes depending on the local near-field enhancement.

The processes of interest for this study are the dissociative ionization of molecules attached to the nanoparticle surface and the ionization of the nanoparticle surface itself. Thus, two near-field-dependent charge distributions are generated in the model: One for the positive, stationary ions resulting from the surface ionization and another one corresponding to free ions resulting from dissociative ionization of adsorbed molecules on the surface. In order to calculate our experimental observable, the final ion momentum, we randomly sample point charges from both distributions and classically propagate the free ions in the electric field of the static ions. The emitted ions are propagated independently of each other with an initial velocity of zero.

The near-field evolution is calculated using the commercially available Lumerical FDTD software (Version 2020a), and the local field enhancement factor $\alpha ({\mathbf {r}})$ around the nanoparticle is extracted as

1 $$\begin{aligned} \alpha ({\mathbf {r}}) = \frac{\max _{t}|{\mathbf {E}}_\text {tot} ({\mathbf {r}},t) |}{\max _{t}|{\mathbf {E}}_\text {in}(t)|}, \end{aligned}$$

where ${\mathbf {E}}_\text {tot}({\mathbf {r}},t)$ is the total electric field around the nanoparticle and ${\mathbf {E}}_\text {in}(t)$ is the electric field of the incident laser pulse. The laser pulse is modeled with a Gaussian temporal envelope with a duration of 25 fs (full width at half maximum of the intensity), and a central wavelength of $2\,\mu$m in agreement with the experimental conditions. In Fig. 2, top row, we show the field enhancement factor $\alpha ({\mathbf {r}})$ in the plane normal to the laser propagation direction containing the center of the nanoparticle. The panels A, C, and E show the field enhancement for different ellipticities.

For the sake of computational efficiency, we approximate the local electric field around the nanoparticle by the incident field rescaled with the local field enhancement factor:

2 $$\begin{aligned} {\mathbf {E}}({\mathbf {r}},t) \approx \alpha ({\mathbf {r}}) {\mathbf {E}}_\text {in}(t). \end{aligned}$$

We tested this approximation by comparing the simulation results for ${\mathbf {E}}({\mathbf {r}},t)$ to the corresponding results for the full FDTD field ${\mathbf {E}}_\text {tot}$. Within our parameter range, we only found negligible differences between both approaches.

As briefly mentioned above, the ionization of the silica nanoparticle is modeled by generating static positive charges on the nanoparticle surface. To be more precise, their spatial distribution $\rho _s({\mathbf {r}})$ is given by:

3 $$\begin{aligned} \rho _s({\mathbf {r}}) \propto 1-\exp \left[ -\int _{-\infty }^\infty \mathrm {d}t\, \gamma ({\mathbf {E}}({\mathbf {r}},t))\right] . \end{aligned}$$

Here, $\gamma$ is the ionization rate, which depends on the local electric field ${\mathbf {E}}({\mathbf {r}},t)$. The ionization rate is calculated using the Ammosov–Delone–Krainov (ADK) formula  [23] applied to the 10.2 eV  ionization potential of silica [24]. The normalization constant of $\rho _s({\mathbf {r}})$ corresponds to the total charge of the nanoparticle, which we estimate from the measured ion energy. The initial distribution of the propagated ions $\rho _p({\mathbf {r}})$ is described similarly to the static charge distribution, yet slightly simpler. As in previous studies, we model the distribution of molecules on the surface as a radial normal distribution centered around the nanoparticle surface, and assume that the ionization probability follows an intensity-dependent power law  [12, 13]:

4 $$\begin{aligned} \rho _p({\mathbf {r}}) \propto |\alpha ({\mathbf {r}})|^{2M} \cdot \exp \left( - \frac{(r-r_0)^2}{2\sigma _r^2} \right) . \end{aligned}$$

The standard deviation $\sigma _r$ of the Gaussian factor is chosen to be 1 nm and its mean $r_0$ is the nanoparticle radius of $150\,$nm. The results are found to be robust with respect to the choice of $\sigma _r$ due to the fast decay of the near-field with increase in distance to the surface. An exponent of $M=I_p/\hbar \omega =21$ was chosen in the power law based on the number of 2 $\mu$m photons required to overcome the ionization potential of the OH radical ($I_p=13.0$ eV)  [25], which is present in silanol groups on silica surfaces (see, for instance, Ref. [26]).

**Fig. 3:**
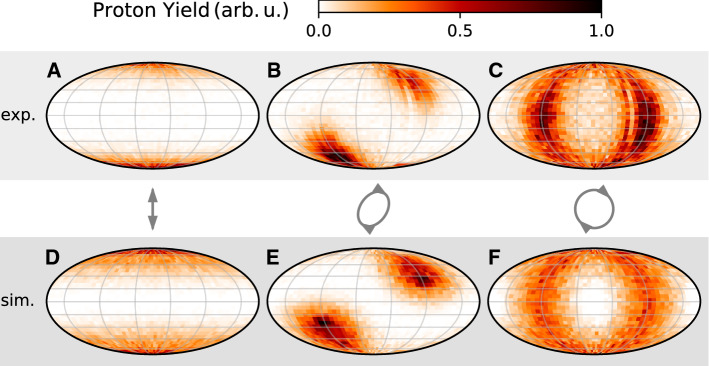
Mollweide projection of the angular distribution of the proton yield from 300 nm silica nanoparticles for a pulse energy of 18 $\mu$J (i.e., $I=2\times 10^{13}$ W/cm$^2$ for lin. pol.). The top row (panels A–C) shows the experimental data. The results of the simulation are displayed in the bottom row (panels D-F). The columns correspond to the same waveplate angles as in Fig. 2 (0$^\circ$, 35$^\circ$, 45$^\circ$) as indicated by the symbols in the middle row

## Results

In order to investigate the applicability of reaction nanoscopy to different wavelengths and polarizations, we compare in Fig. 2 the calculated near-field enhancement to the measured momentum distribution of laser-generated protons. More specifically, the comparison is performed for different (linear, elliptic and circular) polarizations at a wavelength of 2 $\mu$m. The calculated near-field enhancement in the polarization plane is shown in the upper panels. While for linear polarization, we retrieve the same dipolar pattern as in Ref. [12], the field enhancement for circular polarization has a ring-like shape. The corresponding projections of the measured three-dimensional proton momentum distributions onto the polarization plane are shown in the lower panels. From the comparison, a close correspondence between the near-field and ion momentum distributions is observed for the linearly (Fig. 2A,B), elliptically (Fig. 2C,D), and circularly (Fig. 2E,F) polarized pulses, respectively. The striking resemblance between the near-field and the ion spectra suggests that the imaging capability of reaction nanoscopy extends to a wide range of wavelengths and polarizations.

From a theoretical point, the near-field imaging has been successfully modeled using the semi-classical approach described above. The ionization is described by quantum-mechanical ionization and dissociation rates in the local near-field, and ions are propagated as point charges in the electrostatic field of the charged nanoparticle. In order to test the validity of the model for elliptically polarized 2 $\mu$m light, we compare the measured angular distribution of the emitted protons to the model’s prediction in Fig. 3. The comparison shows that the model still provides a good description of the measured angular proton momentum distribution for arbitrary ellipticities. The angular distributions in the simulation are mostly determined by the initial distribution of protons on the surface. In reality, the protons are initially bound in covalently bound surface groups (e.g., OH) or physisorbed solvent molecules (e.g., ethanol) and released from the surface by dissociative ionization. As explained above, the dissociative ionization is modeled by a power law using the ionization potential of OH. While this choice leads to a remarkable agreement in the distributions of Fig. 3, it is based on the assumption that the main source of protons in our experiments are in fact OH groups, i.e., silanols on the silica surface.

**Fig. 4:**
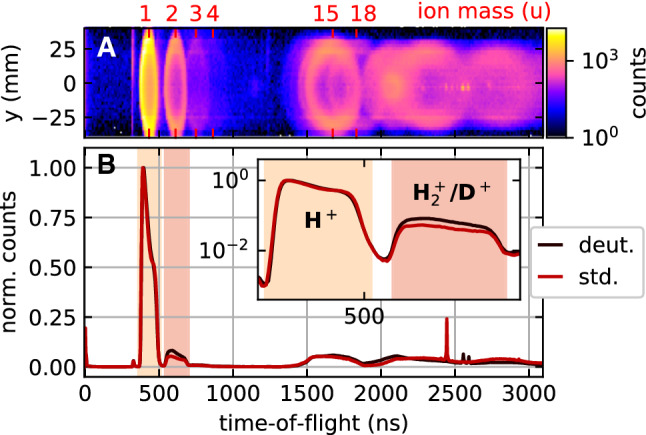
Panel A: Histogram of position vs. time of flight for a measurement of 300 nm silica particles in (fully deuterated) methanol-d4 for circular polarization. Panel B: Time-of-flight spectra of ions emitted from 300 nm silica particles. The particles were aerosolized from dispersions in standard methanol (std.) and methanol-d4 (deut.). Inset: Time-of-flight histograms for ion masses of 1 u and 2 u on a logarithmic scale

In order to provide evidence for our assumption, we investigate the surface composition the silica nanoparticles. In the experiments, the nanoparticles are aerosolized from an alcoholic solution. A silica surface with an adjacent liquid-phase alcohol is well-understood  [27–29]. In such an environment, the silica surface is covered by a mixture of covalently bound OH groups and alkoxy groups. For the case of silica nanoparticles in ethanol, for instance, surface densities of $2.2\pm 0.4$ and $0.5\pm 0.0$ per nm$^2$ were reported for the OH and ethoxy groups, respectively  [30]. In addition, another layer of solvent molecules is physisorbed to this covalent layer via hydrogen bonds  [26, 31]. While we do detect residual amounts of *gas-phase* alcohol in the time-of-flight spectra, the amount of *physisorbed* alcohol molecules on the silica surface is unknown. When trying to remove the solvent alcohol by drying the nanoparticles under ambient conditions, physisorbed molecules remain on the nanoparticles surface and can only be fully removed via a special treatment, for instance by heating the sample to above 100$\circ$C  [30]. This is why, in our case, we expect the hydrogen-bound ethanol to remain on the aerosolized nanoparticles after passing the membrane dryer. However, desorption of the hydrogen-bound ethanol during the final injection of the nanoparticles into the vacuum cannot be excluded. Previous studies found that evacuation does lead, in principle, to desorption of hydrogen-bonded molecules  [27]. On the other hand, the time between the nanoparticles’ injection into the vacuum and their arrival in the interaction region is very short in our experiment (approx. 1 ms). It is, thus, not totally clear to what extent desorption has time to occur within such a brief period.

In order to clarify this question experimentally, we carried out two measurements where we replaced the solvent of the 300 nm particles (ethanol) by methanol and fully deuterated methanol-d4, respectively. Each sample was prepared by two consecutive centrifugation and redispersion cycles following the same protocol as for the cleaning step at the end of the synthesis. The measured time-of-flight spectra for the silica particles aerosolized from a dispersion in methanol and fully deuterated methanol are shown in Fig. 4B. From the striking similarity of the two spectra, we conclude that despite the use of a fully deuterated solvent, the protons still dominate the time-of-flight spectrum. In order to quantify the relative amount of deuterons in the second measurement, we compare the relative amount of ions at mass 1 (H$^+$) and mass 2 (H$^+_2$ and D$^+$) in the spectra (See Fig. 4B, inset). Assuming that the relative increase of the mass 2 signal in the methanol-d4 measurement is solely imputable to D$^+$ ions, we find a value of 0.04 for the ratio of deuterons vs protons for the methanol-d4 case. From this we can clearly conclude that the ion signal from silica nanoparticles in reaction nanoscopy predominantly stems from the covalently bond groups on the surface, while the contribution of physisorbed molecules is negligible. We expect this to be true for any kind of similarly volatile solvent.

Based on this understanding of the surface composition, one would also expect carbon-containing fragments from alkoxy groups in the reaction nanoscopy spectra. We find, indeed, that one of the most abundant ions is CH$_3^+$ (mass 15, Fig. 4A). Similar to the proton/deuteron comparison, the deuterated analogy of CH$_3^+$, namely CD$_3^+$ (mass 18), is much less abundant in the spectrum. Due to the overlapping spectra, however, a quantitative comparison is challenging. Note that we can exclude H$_2$O$^+$ as the source of the ring at mass 18 in Fig. 4A since we only observe this signal with methanol-d4. As one can see as well from the position-resolved time-of-flight spectrum in Fig. 4A, the rings for heavier masses strongly overlap and become less defined, making an analysis of heavier fragments unfeasible. We expect, for example, a contribution of the ethoxy ion and its fragments as well as Si$^+$. Another ion that might be expected is OH$^+$ as the silica surface is covered with a high amount of silanol groups (see above). This fragment, however, is entirely absent from our data. This suggests that upon ionization by the laser, the nanoparticle emits protons and alkyl groups and that the remaining oxygen atoms stay bound with the surface. The exact arrangement and type of the Si-O bonds are an interesting subject for future work but go beyond the scope of the current study.

**Fig. 5:**
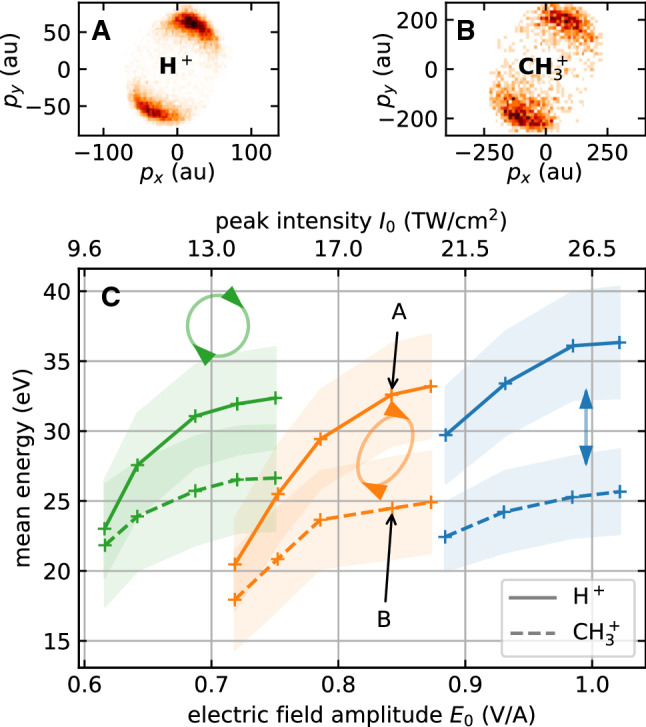
Upper panels: Momentum distributions of H$^+$ (A) and CH$_3^+$ (B) emitted from 300 nm silica nanoparticles. The data was taken at the field amplitude indicated by the corresponding arrows in Panel C. Panel C: Mean ion energies for the same particles as a function of the electric field amplitude $E_0$ and for three polarization states as indicated by the symbols, corresponding to waveplate angles of 45$^\circ$, 30$^\circ$, 0$^\circ$ (left to right). The solid curves show the mean proton energies; the dashed curves show the energies of the CH$_3^+$ ion. The shaded regions represent the width (standard deviation) of the ion energy distribution. The conversion to peak intensity $I_0$ is carried out without cycle-averaging: $I_0=c\varepsilon _0|E_0|^2$

For fragments of different mass but the same charge, such as H$^+$ and CH$_3^+$, our simple static charge model predicts the same final energy as well as similar angular distributions (with slight variations in the width being due to the different ionization potential of different surface groups). However, this significantly departs from the experimental observations. While the angular momentum distributions of different fragments are still strikingly similar (see Fig. 5A,B), the measured ion energies of different masses differ significantly. This can be seen in Fig. 5C, where the intensity-dependent energy of the recorded H$^+$ and CH$_3^+$ ion are plotted for different polarization states. In all cases, the kinetic energy of the CH$_3^+$ ions is suppressed significantly as compared to that of the H$^+$. Moreover, all ion energy curves in Fig. 5 exhibit a similar dependence on the electric field amplitude of the laser field. The ion energies increase linearly at low intensities and flatten out toward higher field strengths. Interestingly, we find that the field amplitude required to generate an ion with a certain energy increases going from circular to linear polarization.

**Fig. 6:**
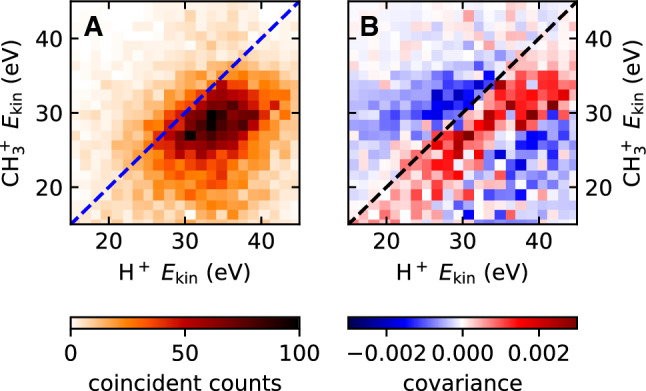
Panel A: Energy correlation histogram of H$^+$ and CH$_3^+$ ions detected in coincidence. Panel B: The covariance map of the same data, defined as: $\text {Cov}(X,Y)_{ij}=\langle X_iY_j \rangle -\langle X_i \rangle \langle Y_j\rangle$, where in our case *X* and *Y* are the kinetic energy of H$^+$ and the CH$_3^+$ and *i*, *j* are the indices of the corresponding energy bins. The angle brackets denote averaging over all laser shots. The dashed lines follow $y=x$

Since the predictive power of the model is limited for the comparison of fragment energies, we will restrict the discussion of the energy ratio of H$^+$ and CH$_3^+$ ions to the experimental aspects. Since CH$_3^+$ is a *molecular* ion, it may undergo dissociation above a certain intensity. In that case, a lower CH$_3^+$ energy would be explicable by a mere focal volume effect. In such a scenario, only low intensity regions in the focal volume would contribute CH$_3^+$ ions. In those regions, the nanoparticle surface would not be as strongly ionized, which in turn leads to a smaller Coulomb repulsion from the surface and an overall smaller CH$_3^+$ energy. We can test whether this focal volume effect plays a role by filtering our experimental data on coincident events, where both, an H$^+$ and a CH$_3^+$ ion were emitted from the same nanoparticle in a single laser shot. In Fig. 6, we show the energy correlation histogram for coincident H$^+$ and CH$_3^+$ events for the linear polarization data of Fig. 5. In 82% of all events, the CH$_3^+$ energy is lower than the H$^+$ energy. We can therefore exclude a focal averaging effect to be responsible for this finding. The same holds true for the other polarization states of Fig. 5 (See supplementary information). Furthermore, we find in Fig. 6 a positive correlation between the energies of H$^+$ and CH$_3^+$, which is especially evident in the covariance map in Fig. 6B.

## Discussion

The fact that the angular ion distribution closely follows the local field enhancement is far from evident. Indeed, it has been shown previously that within the present model, the imaging conditions are not fulfilled, in general, for arbitrary static surface charges distribution   [12]. At a fundamental level, there are two main conditions for near-field imaging. The first condition is that the local yield of an ion species increases monotonically as a function of the electric field. This condition is naturally met by the nonlinear scaling of ionization. It may only be violated at very high field strengths, where saturation and depletion of the singly ionized state become important. The second condition for near-field imaging is that the generated ions are repelled radially from the nanoparticle surface. This condition is only strictly satisfied for a homogeneously charged nanoparticle. However, we find from our model that the similarity between the angular near-field and ion distributions prevails as long as the order of nonlinearity for surface ionization is smaller than for the generation of free ions. In this regime, the surface charge extends over large regions around the points of maximum field enhancement, while the generated ions are much more confined. In this confined area, the field generated by the surface charges repels the ions close-to radially and the imaging conditions are achieved.

Süßmann et al.  [5] reported a close-to linear increase of the electron yield with laser intensity, which is compatible with our result of a linear ion energy increase at low intensity: The higher the number of liberated *electrons*, the higher the effective surface charge of the nanoparticle and, thus, the higher the energy of the repelled *ions*. The saturation-like behavior of the ion energy at high intensity observed in our experiment (see Fig. 5C) is not yet fully understood and requires further investigation.

In principle, the ion energy can serve as a probe of the local and total nanoparticle charge. Assuming that the initial ion momentum is negligible, the final ion energy is completely determined by its initial potential energy. The observed difference in the energy of different ion species, however, proves that this picture is incomplete (See Fig. 5 and 6). According to our present understanding, there are two possible explanations. The first one is simply a difference in the initial energy of different ions. In that case, the energy difference would encode information about the fragments’ binding energies. The other possible reason for the energy differences is a dynamical effect. As positive charges fly away from the nanoparticle surface, the effective surface charge is reduced. Light fragments like protons escape faster than heavier ions like CH$_3^+$. Thus, protons are repelled by the full static charge induced by the laser pulse, while the CH$_3^+$ ions experience a reduced charge, once the protons have escaped from the nanoparticle. This effect may explain the gradual decrease of the ring size with increase in time of flight in Fig. 4A, which directly corresponds to a decrease in kinetic energy with increase in ion mass. However, further studies are required to fully elucidate the contributions to the fragments kinetic energies.

## Conclusions

In conclusion, we have shown that the concept of near-field imaging by ions emitted from a strong-field ionized nanoparticle surface extends toward the mid-infrared wavelength regime and to an arbitrary ellipticity of the laser polarization. We found that a theoretical description in terms of a semi-classical model provides a good qualitative description of the near-field imaging. A quantitative prediction of the ion kinetic energies, however, will require a more elaborate theoretical treatment.

Furthermore, we have clarified the surface composition of the aerosolized silica nanoparticles, which had not yet been addressed systematically in previous reaction nanoscopy studies. In particular, we have shown that the recorded signal is dominated by protons from covalently bound silanol groups, while the contribution of the solvent (alcohol) is rather negligible. These findings not only settle an important open question about the origin of the measured signal. They also provide the experimental basis for the development of a more accurate theoretical treatment of nanoparticle dynamics in strong laser fields.

## Supplementary Information

Below is the link to the electronic supplementary material.

Supplementary file 1 (pdf 2690 KB)

## Data Availability

This manuscript has no associated data or the data will not be deposited. [Authors’ comment: The datasets generated during and/or analyzed during the current study are available from the corresponding author on reasonable request.]
