# Supplementary material for: Imaging elliptically polarized infrared near-fields on nanoparticles by strong-field dissociation of functional surface groups
Source: Eur Phys J D At Mol Opt Phys. 2022 Jun 27;76(6):109. doi: 10.1140/epjd/s10053-022-00430-6 (PMC9236975; doi:10.1140/epjd/s10053-022-00430-6)
Supplement: Supplementary file 1 — (pdf 2690 KB) [file 10053_2022_430_MOESM1_ESM.pdf]

Supplementary Information

Imaging elliptically polarized infrared near-fields on nanoparticles by strong-field dissociation of functional surface groups

Philipp Rosenberger[1,2], Ritika Dagar[1,2], Wenbin Zhang[1,2,3], Ana Sousa-Castillo[4], Marcel Neuhaus[1,2], Emiliano Cortes[4], Stefan A. Maier[4,5], Cesar Costa-Vera[6], Matthias F. Kling[1,2,7,8*] and Boris Bergues[1,2*]

[1]Department of Physics, Ludwig-Maximilians-Universität Munich, D-85748 Garching, Germany.
[2]Max Planck Institute of Quantum Optics, D-85748 Garching, Germany.
[3]State Key Laboratory of Precision Spectroscopy, East China Normal University, Shanghai 200241, China.
[4]Chair in Hybrid Nanosystems, Nanoinstitute Munich, Königinstrasse 10, Faculty of Physics, LMU Munich, 80539 Munich.
[5]Department of Physics, Imperial College London, London SW7 2AZ, UK.
[6]Departamento Fisica, Escuela Politecnica Nacional, 170109 Quito, Ecuador.
[7]SLAC National Accelerator Laboratory, Menlo Park, CA 94025, USA.
[8]Applied Physics Department, Stanford University, Stanford, CA 94305, USA.

*Corresponding author(s). E-mail(s): kling@stanford.edu; boris.bergues@mpq.mpg.de;

1 Size distribution of the silica nanoparticles

We determined the size distribution of the silica nanoparticles from a representative SEM using a combinations of algorithms from the openCV library. The green rectangles of Figure S1 A indicate the detected nanoparticles. The size distribution of all detected nanoparticles is displayed in Figure S1 B. The standard deviation of the size distribution is about 6%.

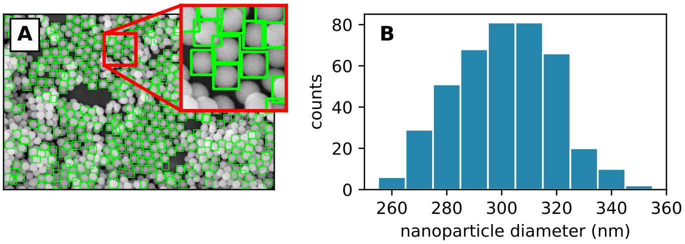

Fig. S1 Panel A: SEM image of the silica nanoparticles used for this study. The green rectangles mark the particles that were detected by the software. Panel B: The histogram of the particle diameters from all marked particles of panel A.

2 Energy correlation between H^+ and CH_3^+

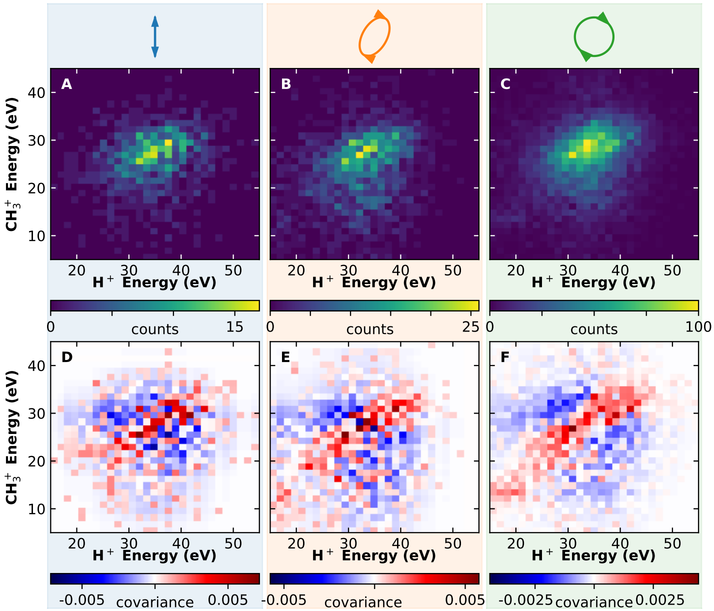

Fig. S2 Energy correlation between H^+ and CH_3^+ for different polarization states. The top row shows the histograms of coincident events. The bottom row shows the corresponding covariance maps. See the main text for the definition.
